# A chimeric trivalent Echovirus vaccine designed by loop substitution elicits cross-neutralizing immunity against serotypes 11, 18, and 30

**DOI:** 10.1128/jvi.00457-26

**Published:** 2026-05-05

**Authors:** Shuai Jiang, Jiaqi Cui, Tao Sun, Jiahuang Li, Heming Li, Tianlun Chen, Jiahui Wu, Zhenhua Zheng, Jianbo Xia, Chunchen Wu, Jie Wu

**Affiliations:** 1 School of Life Science and Technology, China Pharmaceutical University 56651, Nanjing, China; 2 School of Biopharmacy, China Pharmaceutical University, Nanjing, China; 3 CAS Key Laboratory of Special Pathogens and Biosafety, Center for Emerging Infectious Diseases, Wuhan Institute of Virology, Chinese Academy of Sciences 74614 , Wuhan, China; 4 Department of Laboratory Medicine, Maternal and Child Health Hospital of Hubei Province, Tongji Medical College, Huazhong University of Science and Technology 12443, Wuhan, China; University of Kentucky College of Medicine, Lexington, Kentucky, USA

**Keywords:** Echovirus, trivalent vaccine, loop substitution, mRNA vaccine, Enterovirus

## Abstract

Echoviruses, members of the Enterovirus B genus in the Picornaviridae family, comprise numerous serotypes that frequently cause aseptic meningitis, myocarditis, and severe neonatal infections. The coexistence of multiple antigenically distinct serotypes poses a significant challenge for vaccine development, underscoring the clinical need for a broad-spectrum multivalent vaccine. In this study, we focused on three prevalent serotypes—Echovirus 11 (E11), 18 (E18), and 30 (E30)—as representative models to explore multivalent vaccine design. We first predicted and experimentally identified dominant B-cell epitopes within the VP1 proteins of these viruses. Guided by these analyses, a chimeric trivalent antigen (VP1-III) was rationally constructed by substituting epitope-containing loops from E18 and E30 into the VP1 backbone of E11. Structural modeling confirmed that VP1-III preserved native VP1 conformation while displaying multitype epitopes. Both VP1-III recombinant protein and mRNA-lipid nanoparticle vaccines induced potent immunogenicity in mice, characterized by cross-neutralizing antibodies and Th1-biased cellular responses. These findings support structure-guided epitope integration as a promising strategy for developing broadly protective multivalent Echovirus vaccines.

**IMPORTANCE:**

Echoviruses cause widespread outbreaks and severe neonatal disease, yet vaccine development remains limited by extensive antigenic diversity among serotypes. Here, we rationally engineered a chimeric trivalent VP1 antigen by grafting dominant epitopes from E11, E18, and E30 through structure-guided loop substitution. Both recombinant protein and mRNA formulations induced robust cross-neutralizing and Th1-biased immune responses in mice, demonstrating that strategic epitope substitution can effectively overcome serotype barriers and providing a promising path toward broad-spectrum Echovirus vaccines.

## INTRODUCTION

Echoviruses, members of the genus Enterovirus B (EV-B) in the family Picornaviridae, are non-enveloped RNA viruses that cause a broad spectrum of diseases, including aseptic meningitis, myocarditis, and acute flaccid paralysis (1). In severe cases, infection can result in hemorrhage, shock, multiple organ failure, and even death (2). Despite their clinical significance, no licensed vaccine is currently available for Echovirus infections, and treatment remains largely supportive (3, 4). The absence of effective prophylactic measures not only facilitates viral transmission but also poses a substantial threat to high-risk populations, particularly neonates and young infants (5).

To date, about 30 Echovirus serotypes have been identified, with E11, E18, E30, and E9 most frequently linked to human outbreaks (6). Limited cross-neutralization among serotypes represents a major challenge for vaccine development. Therefore, the development of a broadly protective multivalent vaccine capable of preventing infections caused by multiple Echovirus serotypes is of critical public health and clinical relevance (7).

The icosahedral capsid of Echovirus, about 30 nm in diameter, is assembled from 60 protomers, each consisting of four structural proteins (VP1–VP4) arranged into 12 pentameric units (8). VP1–VP3 are exposed on the virion surface, whereas VP4 is located internally, and all serotypes share a conserved overall architecture (9). VP1, the most surface-exposed and immunodominant protein, serves as the principal determinant of viral neutralization and thus represents a key target for vaccine design (10, 11).

Protein-based vaccines can directly deliver purified antigens formulated with adjuvants, mainly relying on exogenous antigen presentation and adjuvant-mediated activation (12, 13). In contrast, mRNA vaccines elicit stronger humoral and cellular immune responses by simulating endogenous antigen expression and activating MHC-I and MHC-II presentation pathways (14). mRNA vaccination typically induces higher antibody titers and more balanced Th1/Th2 responses without the need for traditional adjuvants (15). Therefore, mRNA vaccines typically provide broader and more durable protection against viral infections, although protein-based vaccines still have advantages in production stability and long-term safety validation (16).

In this study, B-cell epitopes of E11-VP1, E18-VP1, and E30-VP1 were predicted using bioinformatics approaches. Dominant antigenic epitopes were identified through enzyme-linked immunosorbent assay (ELISA)-based screening using VP1-specific antisera. And then, a loop substitution strategy was employed to construct a chimeric trivalent antigen, termed VP1-III, which incorporated representative epitopes from the three serotypes. Furthermore, an mRNA vaccine encoding VP1-III was also developed. Both VP1-III and VP1-III-mRNA were evaluated for their ability to elicit cross-reactive antibody responses. These findings establish a rational framework for the development of broadly protective multivalent vaccines against neonatal Echovirus infections.

## RESULTS

### Prediction and identification of antigenic epitopes in E11, E18, and E30 VP1 proteins

**Fig 1:**
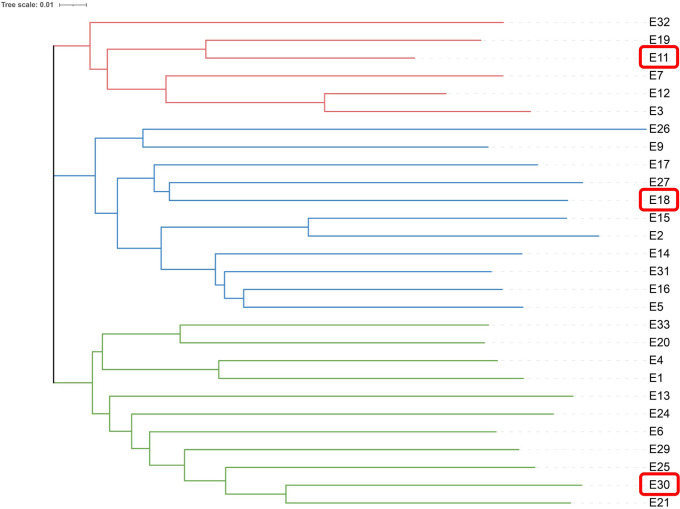
Phylogenetic construction of Echovirus. E11, E18, and E30 were located on different branches in phylogenetic analysis based on VP1 sequences.

To identify conserved and immunodominant epitopes suitable for multivalent vaccine design, we first performed a systematic prediction and experimental validation of B-cell epitopes in the VP1 proteins of E11, E18, and E30. Phylogenetic analysis encompassing 28 echovirus serotypes revealed that E11, E18, and E30 each reside on distinct evolutionary branches (Fig. 1). Thus, developing a trivalent vaccine targeting these three serotypes may provide broad protection against the prevalent Echovirus and may reduce overall transmission. Given that VP1 is the principal antigenic capsid protein and a major target for neutralizing antibodies, immunoinformatic tools were employed to predict linear B-cell epitopes for chimeric vaccine design. Using ABCpred, BCPreds, and Bepipred Linear Epitope Prediction 2.0, 9, 12, and 9 B-cell epitopes were identified in E11-VP1, E18-VP1, and E30-VP1, respectively (Table S1). After merging overlapping sequences, six representative epitopes were obtained for each serotype, E11-Peptide 1–6, E18-Peptide 1–6, and E30-Peptide 1–6 (Table 1).

**TABLE 1:** Synthesized B-cell epitopes based on amino acid sequences and structure

Name	Sequence	Start	End	MW (Da)	pI	GRAVY
E11-Peptide 1	TFVITSKQDQGTQLGQ	120	135	1,750.93	5.50	−0.613
E11-Peptide 2	PPLTHQVMYIPPGGPIPKSTTDYAWQTSTNPSIFWTE	138	174	4,159.68	5.33	−0.530
E11-Peptide 3	AYSNFYDGWSHFSQNGVYGYNTLNN	191	215	2,919.03	5.08	−0.908
E11-Peptide 4	TLNNMGQLYMRHVNGP	212	227	1,845.12	8.44	−0.656
E11-Peptide 5	NGPSPLPMTSTVRVYF	225	240	1,766.04	8.75	−0.037
E11-Peptide 6	CQYINAPTVNFSSTNI	256	271	1,771.96	5.52	0.025
E18-Peptide 1	NFMGRAACVFMDQYKLNGEETSTDNFAVWT	60	89	3,446.83	4.32	−0.333
E18-Peptide 2	CQDQGTQLEQDMPVLT	121	136	1,805.99	3.49	−0.731
E18-Peptide 3	PVLTHQIMYVPPGGPIPAKVDSYEWQTSTNPSVFWTE	132	169	4,173.71	4.65	−0.324
E18-Peptide 4	TTLNAMGKLFVRHVNK	206	221	1,829.19	11.17	−0.113
E18-Peptide 5	SSPHQITSTIRVYFKP	222	237	1,861.13	9.99	−0.463
E18-Peptide 6	PRPPRLCPYINKGDVNFVVTEVT	245	267	2,615.05	8.60	−0.261
E30-Peptide 1	EKVNDELDRYTNWEIT	82	97	2,025.16	4.18	−1.500
E30-Peptide 2	NWEITTRQVAQLRRKL	93	108	2,012.35	11.71	−0.975
E30-Peptide 3	TSSQRTSTTYASDSPP	125	140	1,685.72	5.50	−1.312
E30-Peptide 4	PPLTHQVMYVPPGGPIPKSYEDFAWQTSTNPSVFWTE	139	175	4,205.71	4.65	−0.527
E30-Peptide 5	PKHVKAWVPRAPRLCP	243	258	1,855.28	11.02	−0.562
E30-Peptide 6	PYLYARNVNFDVQGVT	258	273	1,856.07	6.26	−0.175

**Fig 2:**
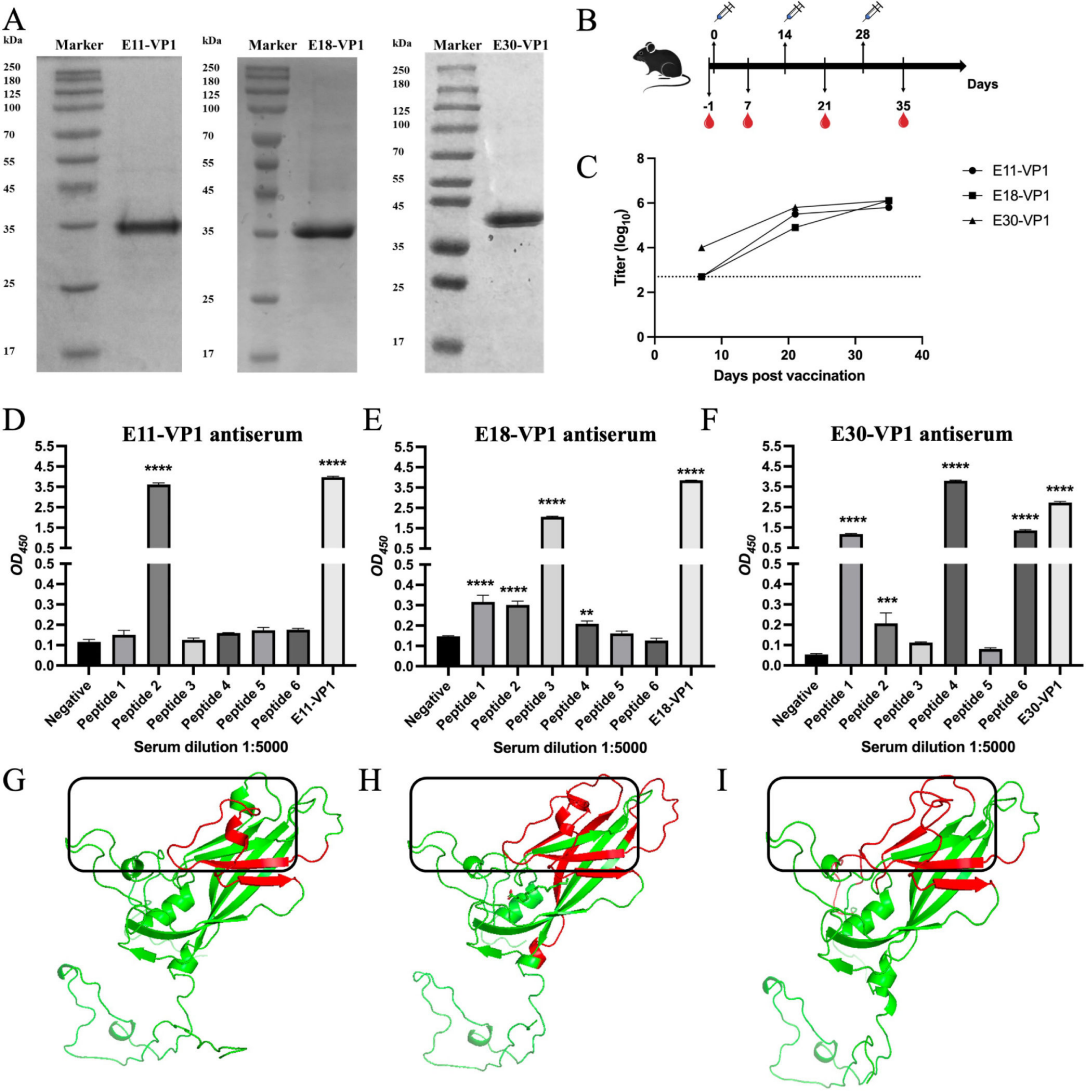
Mouse anti-E11/18/30-VP1 serum was collected and used to screen B-cell epitopes. (**A**) SDS-PAGE of E11-VP1, E18-VP1, and E30-VP1. (**B**) Immune schedule of mice. (**C**) Changes of antibody titers during the immunization induced by E11/18/30-VP1 were detected by ELISA. The dashed line represents the lower limit of detection (sera were collected before immunization). Binding of the epitopes with E11-VP1 (**D**), E18-VP1 (**E**), and E30-VP1 (**F**) antiserum detected by ELISA. Screened epitopes in the structure of E11-VP1 (**G**), E18-VP1 (**H**), and E30-VP1 (**I**). The receptor-binding regions are highlighted and enclosed by black boxes. E11-VP1, E18-VP1, and E30-VP1 were colored green, epitopes were colored red. **P < 0.01; ***P < 0.001; ****P < 0.0001.

Recombinant E11-VP1, E18-VP1, and E30-VP1 proteins were expressed in *Escherichia coli* BL21(DE3) and purified (Fig. 2A). Mice immunized subcutaneously with these proteins using Freund’s adjuvant (0.1 mg antigen per mouse) exhibited increasing antibody titers, with significant elevation after the second and third immunizations (Fig. 2B and C). Binding of peptides to their corresponding sera revealed that E11-Peptide 2, E18-Peptides 1/2/3/4, and E30-Peptides 1/2/4/6 were strongly recognized (Fig. 2D through F). The locations of these reactive epitopes were mapped onto the structural schematic of the VP1 protein (Fig. 2G through I).

**Fig 3:**
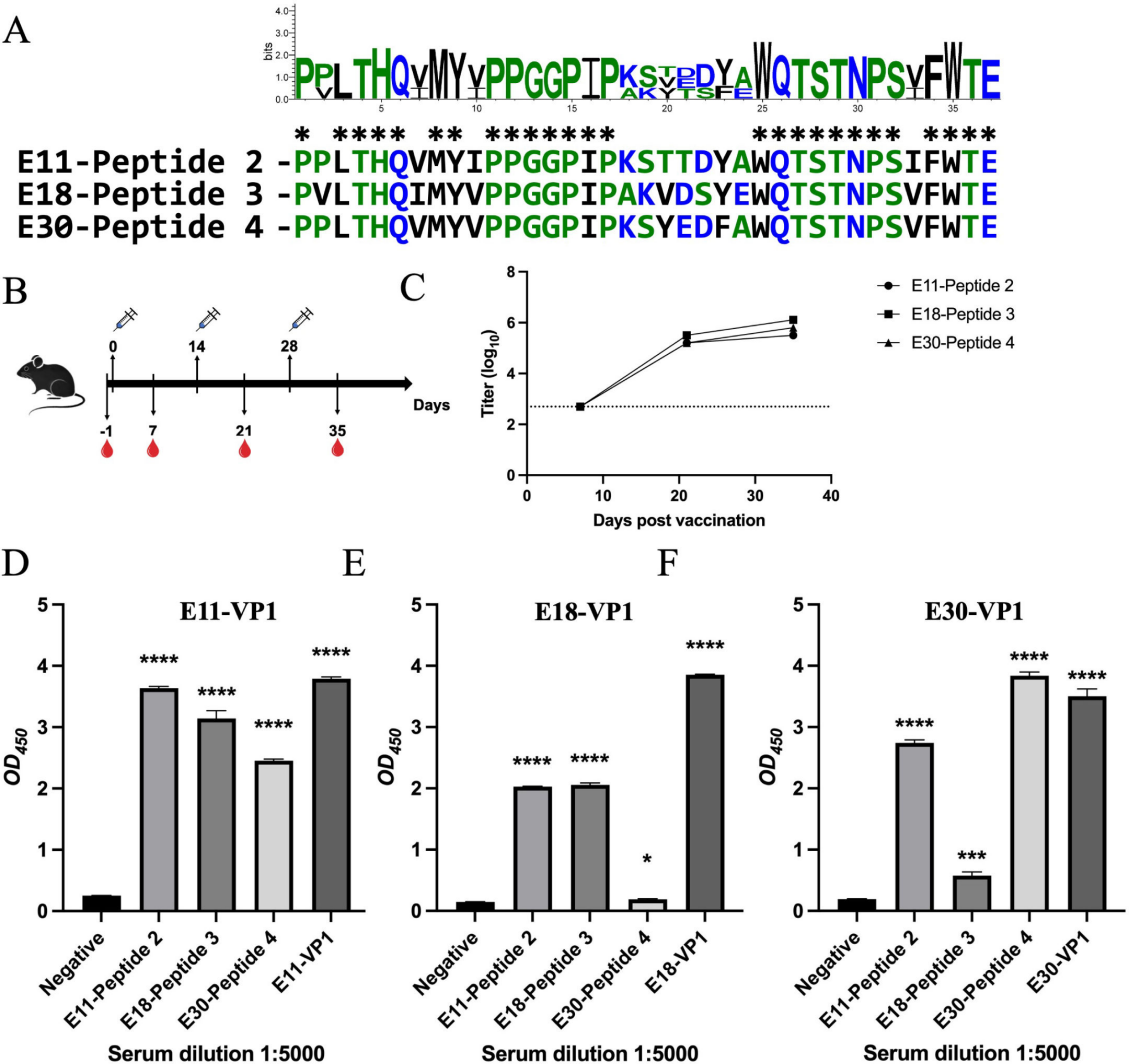
E11-Peptide 2, E18-Peptide 3, and E30-Peptide 4 were immunized and showed overlapping binding ability. (**A**) Sequence logo and sequence alignments of E11-Peptide 2, E18-Peptide 3, and E30-Peptide 4 were obtained from WebLogo 3 and MEGA 11. (**B**) Immune schedule of mice. (**C**) Changes of antibody titers during the immunization induced by E11-Peptide 2, E18-Peptide 3, and E30-Peptide 4 were detected by ELISA. The dashed line represents the lower limit of detection (sera were collected before immunization). Binding of the E11-Peptide 2, E18-Peptide 3, E30-Peptide 4, or VP1s antiserum with E11-VP1 (**D**), E18-VP1 (**E**), and E30-VP1 (**F**) detected by ELISA. *P < 0.05; ***P < 0.001; ****P < 0.0001.

Among them, E11-Peptide 2, E18-Peptide 3, and E30-Peptide 4 showed the strongest binding ability to the antigen and exhibited a high degree of homology (Fig. 3A). To evaluate their potential for cross-reactivity, these three long peptides were used to immunize mice. Antibody titers increased significantly by day 14 and peaked by day 35 (Fig. 3B and C). ELISA results demonstrated strong binding of all three antisera to E11-VP1 (Fig. 3D). Antisera raised against E11-Peptide 2 and E18-Peptide 3 also recognized E18-VP1 (Fig. 3E), whereas the antiserum directed against E30-Peptide 4 exhibited comparatively weaker binding. In Fig. 3F, E11-Peptide 2 and E30-Peptide 4 antisera reacted with E30-VP1, while the E18-Peptide 3 antiserum exhibited substantially reduced binding in comparison. Notably, E11-Peptide 2 displayed robust reactivity across all three VP1 antigens, suggesting its potential as a conserved cross-reactive epitope for multivalent vaccine development.

### Design of the trivalent chimeric vaccine VP1-III

**Fig 4:**
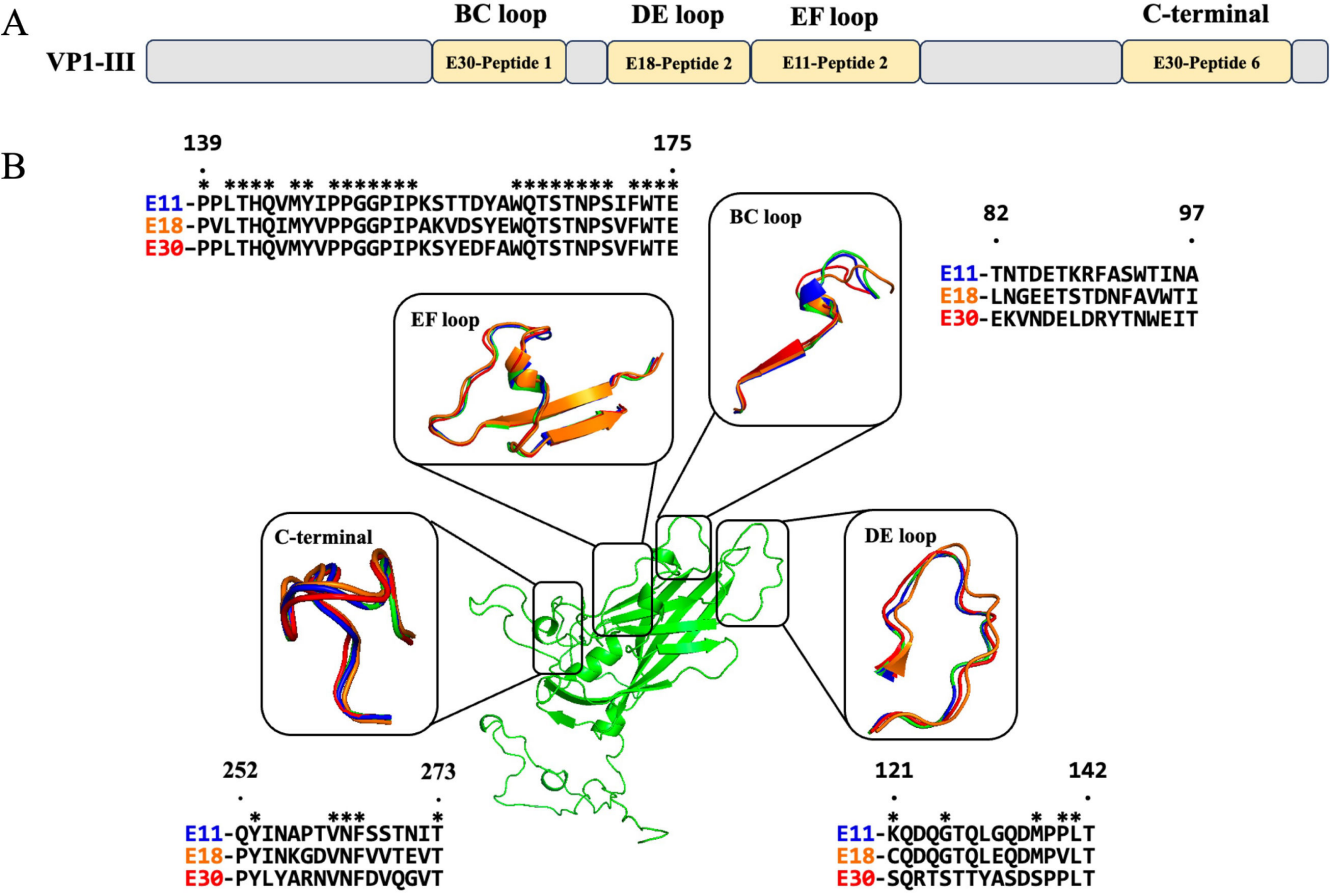
A trivalent chimeric vaccine (VP1-III) was designed by inserting E30-Peptide 1, E18-Peptide 2, and E30-Peptide 6 into E11-VP1, and the structure was predicted. (**A**) Design of the vaccine, epitopes were shown as yellow modules. (**B**) Prediction model of VP1-III structure, in which inserted epitopes were compared among E11/18/30 peptides. The region spanning amino acids 82–97 of E30-Peptides 1, 121–142 of E18-Peptides 2, 143–175 of E11-Peptide 2, and 252–273 of E30-Peptides 6 was selected for substitution in the chimeric VP1 construct. VP1-III, E11-VP1, E18-VP1, and E30-VP1 were colored green, blue, orange, and red, respectively.

To translate the identified epitopes into a practical immunogen, we next designed a trivalent chimeric VP1 antigen incorporating the most immunogenic and cross-reactive regions from E11, E18, and E30. Based on the predicted linear B-cell epitopes, a trivalent chimeric VP1 antigen (VP1-III) was designed using loop replacement. E11-VP1 was selected as the structural backbone due to its epidemiological predominance and the presence of a conserved, cross-reactive epitope (E11-Peptide 2). Epitopes from E18 and E30 were substituted into corresponding loop regions of E11-VP1: E30-Peptide 1 replaced the BC loop, E18-Peptide 2 replaced the DE loop, and E30-Peptide 6 was inserted at the C-terminal region. The resulting construct contained four key epitopes, E11-Peptide 2, E18-Peptide 2, E30-Peptide 1, and E30-Peptide 6 (Fig. 4A). The SWISS-MODEL-generated structure of VP1-III suggested that the introduced epitopes could be integrated into surface-exposed loops without substantially altering the canonical VP1 β-barrel fold. Structural superposition with E11-, E18-, and E30-VP1 highlighted local variations—most prominently in the BC loop—that may underlie differences in serotype-specific antigenicity (Fig. 4B).

### VP1-III and VP1-III mRNA vaccines induced cross-reactive antibody responses in mice

**Fig 5:**
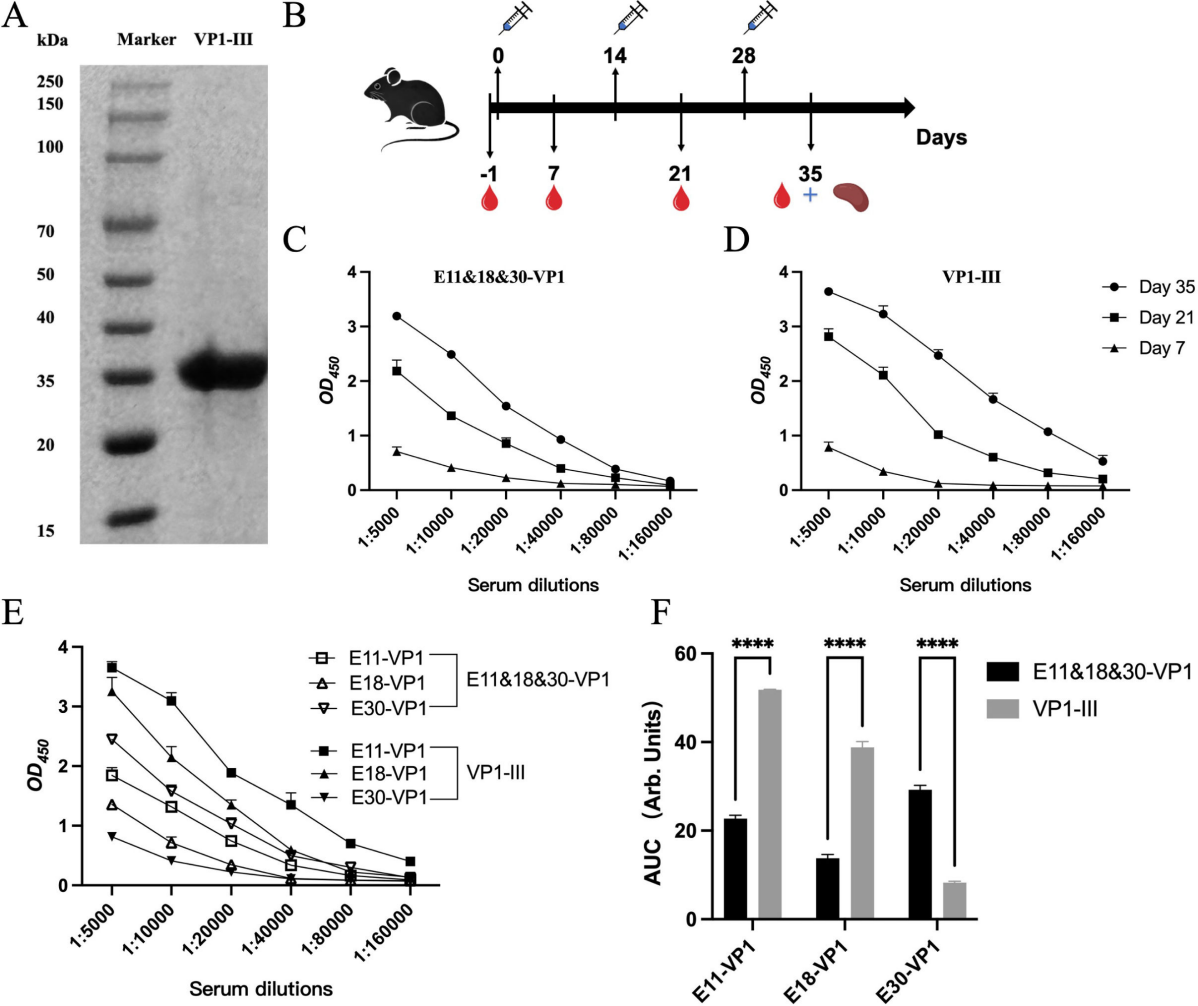
VP1-III was expressed, and the antiserum titer was obtained to study humoral immunity. (**A**) SDS-PAGE of VP1-III. (**B**) Immunization schedule of mice. (**C and D**) Changes in antibody titers during the immunization induced by E11&18&30-VP1 and VP1-III were detected by ELISA, respectively. (**E**) Binding of E11&18&30-VP1 and VP1-III antiserum with E11-VP1, E18-VP1, and E30-VP1 detected by ELISA, respectively. (**F**) Area under the curve (AUC) analysis of three experimental groups represented in panel E. Arb. Units, arbitrary units. ****P < 0.0001.

We next evaluated the immunogenicity of VP1-III to determine its ability to elicit cross-binding activity across the three Echovirus serotypes. To directly compare with a conventional multivalent vaccine strategy, an E11&18&30-VP1 control group was included, in which recombinant E11-VP1, E18-VP1, and E30-VP1 were mixed at equal doses. Recombinant VP1-III protein was successfully expressed and purified (Fig. 5A). Mice were immunized subcutaneously with VP1-III or E11&18&30-VP1 mixture formulated in Freund’s adjuvant. Both immunogens induced robust and gradually elevated serum antibody titers, which peaked after the third immunization (Fig. 5B through D). ELISA analysis demonstrated that antisera from both the VP1-III and E11&18&30-VP1 groups exhibited cross-reactivity with all three VP1 antigens, indicating that VP1-III successfully elicited broadly cross-reactive antibodies comparable to those induced by the mixed-VP1 immunization (Fig. 5E and F). Notably, the E11&18&30-VP1 mixture showed significantly weaker binding to E11-VP1 and E18-VP1 compared with VP1-III, but significantly stronger binding to E30-VP1. These results indicate that both immunization strategies elicited skewed antibody responses, but with different antigenic preferences. Collectively, these data demonstrate that the chimeric VP1-III, which combines epitopes from three VP1 proteins, can induce cross-neutralizing antibodies with a breadth similar to that of the mixed VP1 immunogen.

**Fig 6:**
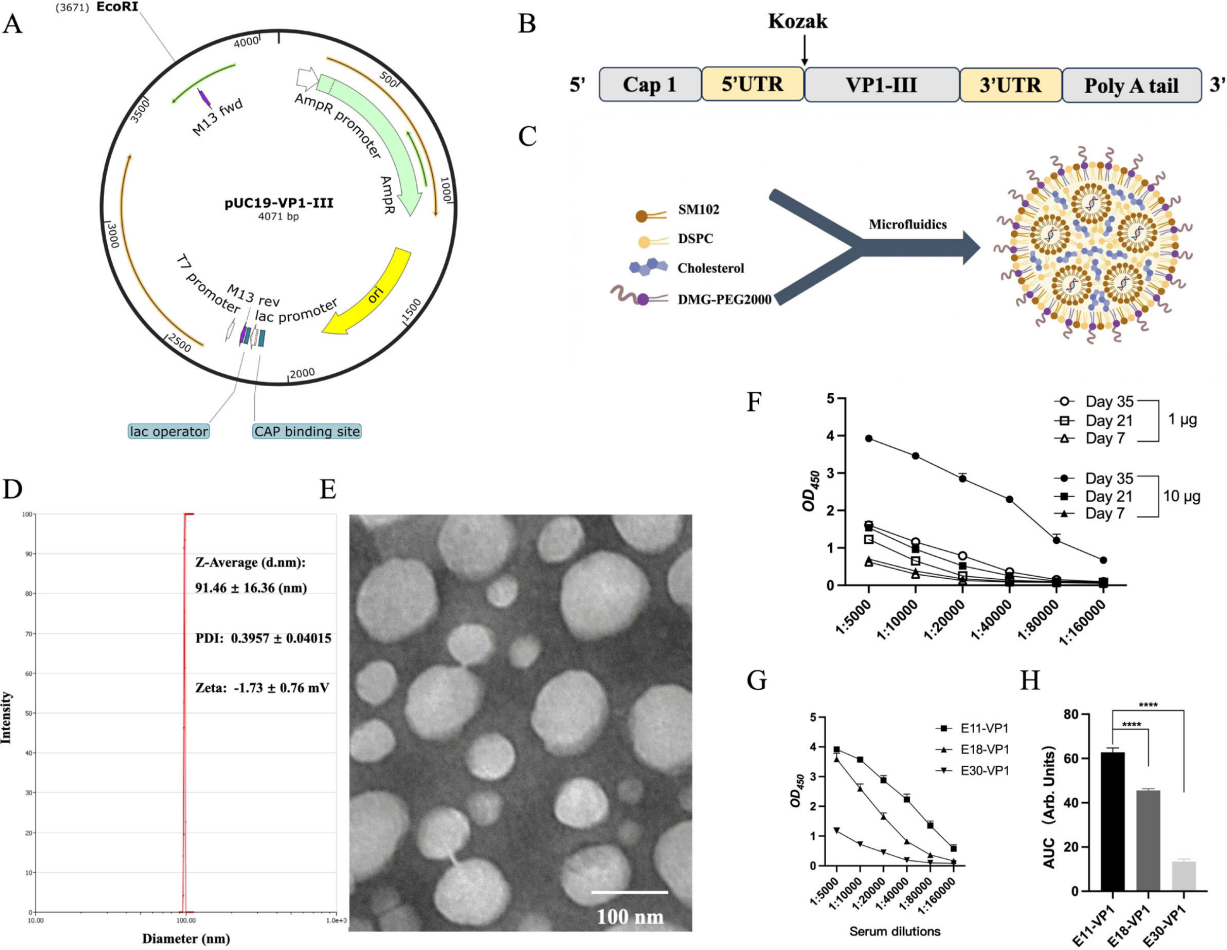
VP1-III-mRNA was designed and produced, and the antiserum titer was obtained to study humoral immunity. (**A**) Schematic diagram of plasmids pUC19-VP1-III. (**B**) Schematic diagram of mRNA structure. (**C**) Schematic illustration of the mRNA-LNP formulation process. (**D**) Particle size graph and zeta potential of mRNA-LNP. (**E**) TEM of mRNA-LNP. (**F**) Changes in antibody titers during the immunization induced by 1 and 10 µg VP1-III-mRNA detected by ELISA. (**G**) Binding of VP1-III-mRNA antiserum with E11-VP1, E18-VP1, and E30-VP1 detected by ELISA. (**H**) AUC of three experimental groups represented in panel G. ****P < 0.0001.

Furthermore, VP1-III mRNA was synthesized and encapsulated into lipid nanoparticles (LNPs). pUC19-VP1-III, containing a 5′UTR, VP1-III coding sequence, 3′UTR, and a 100 nt poly(A) tail was designed and constructed (Fig. 6A and B). *In vitro* transcription (IVT) and capping were performed using the purified plasmids as templates. mRNA-LNP was synthesized via microfluidic mixing and characterized by hydrodynamic diameter, PDI, zeta potential, and encapsulation efficiency. The LNPs exhibited an encapsulation efficiency of 97.0 ± 0.37%, particle size of 91.5 ± 16.4 nm, and zeta potential of −1.7 ± 0.8 mV, and transmission electron microscopy (TEM) revealed spherical LNPs consistent with the DLS data (Fig. 6C through E). Mice immunized intramuscularly with low (1 μg) or high (10 μg) doses of VP1-III-mRNA showed dose-dependent antibody responses, with significantly stronger antigen binding in the high-dose group after the third immunization (Fig. 6F). In contrast, the low-dose group failed to induce highly binding antibodies in mice; therefore, the high-dose group was used in subsequent experiments. Similar to the VP1-III protein group, sera from mice immunized with VP1-III-mRNA showed robust binding to all three serotype VP1 proteins, exhibiting the highest binding affinity to E11-VP1, with lower reactivity to E18-VP1 and E30-VP1 (Fig. 6G and H).

### VP1-III and VP1-III mRNA vaccines elicited strong cross-neutralizing sera

**Fig 7:**
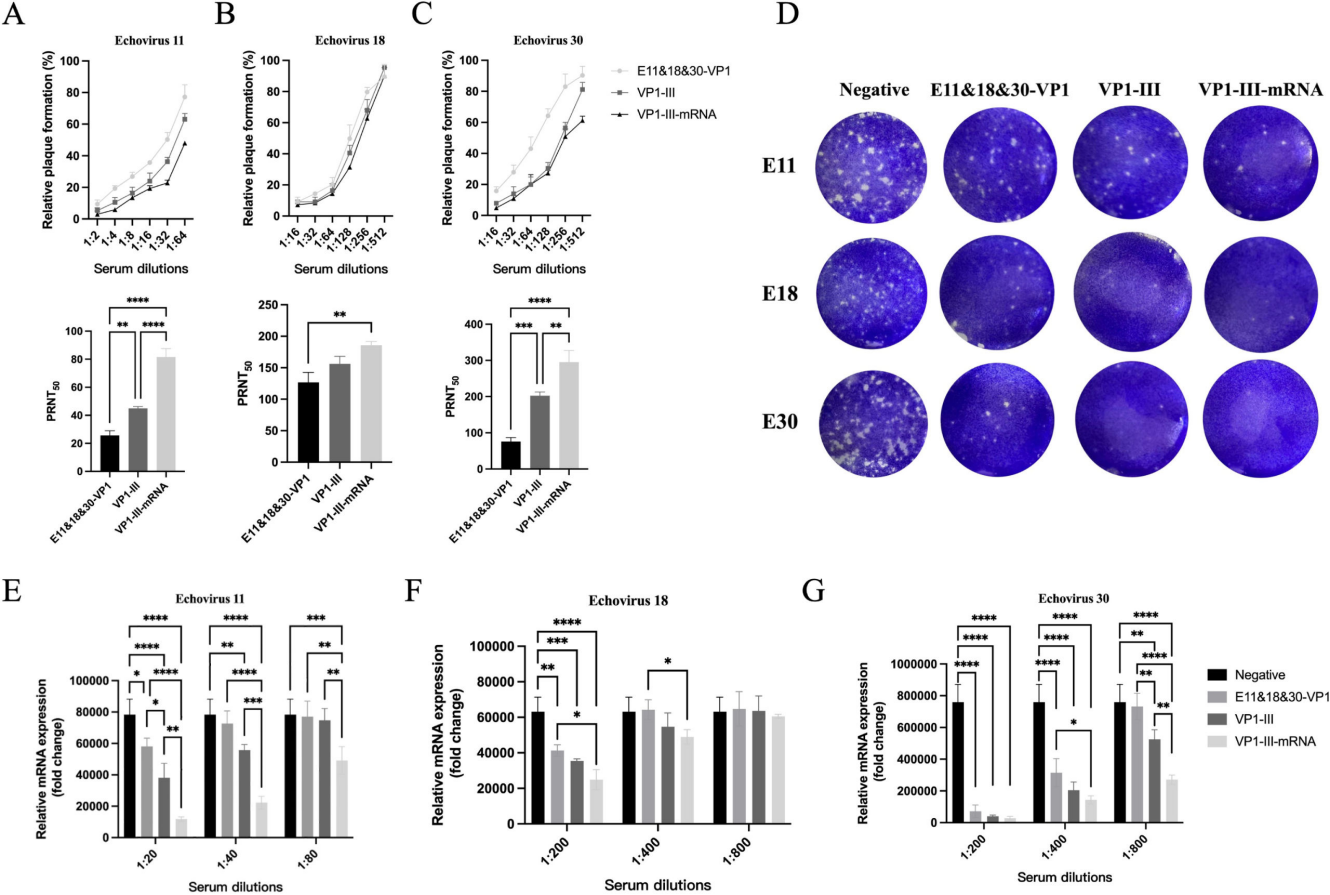
Immunogenicity of VP1-III and VP1-III-mRNA. Neutralization of E11 (**A**), E18 (**B**), and E30 (**C**) viruses by E11&18&30-VP1, VP1-III, and VP1-III-mRNA antisera was detected by relative plaque formation and plaque reduction neutralization test 50 (PRNT_50_). (**D**) Examples of plaque counting. Antiserums of E11&18&30-VP1, VP1-III, and VP1-III-mRNA were diluted at a ratio of 1:32. Neutralization of E11 (**E**), E18 (**F**), and E30 (**G**) viruses by antisera, as determined by viral RNA quantification. *P < 0.05; **P < 0.01; ***P < 0.001; ****P < 0.0001.

To determine the antibody responses induced by VP1-III and VP1-III-mRNA vaccines, we next assessed their neutralizing activity against the three Echovirus serotypes. Plaque reduction neutralization tests (PRNTs) showed that sera from all groups effectively neutralized all three Echovirus serotypes. For E11 and E30, both VP1-III and VP1-III-mRNA induced significantly higher neutralizing activity than E11&18&30-VP1 (Fig. 7A and C). VP1-III-mRNA elicited PRNT_50_ titers of 81.53 and 295.3 against E11 and E30, respectively, representing approximately 1.8-fold and 1.5-fold increases compared with VP1-III. For E18, VP1-III-mRNA induced significantly higher neutralizing titers than the E11&18&30-VP1 group, whereas VP1-III showed no statistically significant difference from the E11&18&30-VP1 control (Fig. 7B). Representative plaque reduction images corroborate these observations (Fig. 7D). Interestingly, this neutralization pattern was not entirely consistent with the ELISA binding results, as the E11&18&30-VP1 group showed significantly stronger binding to E30-VP1 than that of VP1-III. These findings suggest that some binding epitopes detected by ELISA may not correspond to neutralizing epitopes.

Quantitative RT-PCR further substantiated the neutralization data. For E11, at a serum dilution of 1:20, VP1-III-mRNA exhibited significantly stronger neutralizing activity than the VP1-III and E11&18&30-VP1, and VP1-III also exhibited significantly stronger activity than E11&18&30-VP1 (Fig. 7E). At higher dilutions (1:40 and 1:80), VP1-III-mRNA remained significantly superior, while VP1-III and E11&18&30-VP1 showed no significant difference. For E18, no significant differences were observed between VP1-III and the E11&18&30-VP1 group at dilutions of 1:200, 1:400, and 1:800; however, VP1-III-mRNA demonstrated significantly enhanced neutralization at 1:200 and 1:400 compared with E11&18&30-VP1 (Fig. 7F). For E30, VP1-III exhibited significantly stronger neutralization than the E11&18&30-VP1 group only at a dilution of 1:800, whereas VP1-III-mRNA showed significantly stronger neutralizing activity at both 1:400 and 1:800, and surpassed VP1-III at the highest dilution tested (Fig. 7G). Collectively, these data indicate that VP1-III induces broader and more potent neutralizing responses than a simple mixture of individual VP1 antigens, with further enhancement achieved through mRNA delivery.

### VP1-III and VP1-III mRNA vaccines induce potent cellular immune responses

**Fig 8:**
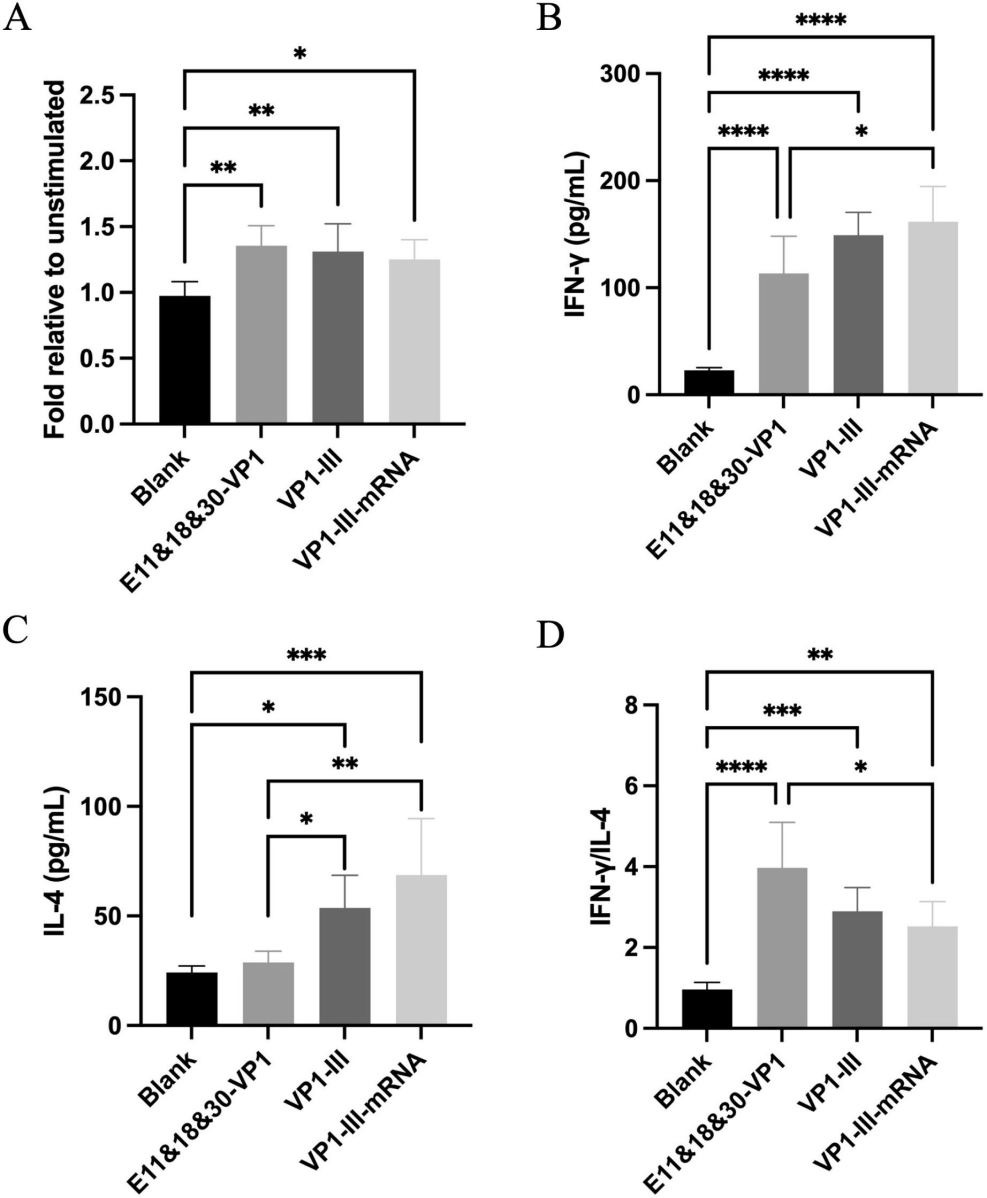
VP1-III and VP1-III-mRNA induced potent cell immunity. Splenocytes were isolated from immunized mice at day 35 post initial immunization and stimulated with the corresponding epitope peptides. Antigen-specific cellular immune responses were assessed by measuring (**A**) splenocyte proliferation, (**B**) IFN-γ secretion, (**C**) IL-4 secretion, and the (**D**) IFN-γ/IL-4 ratio in mice immunized with E11&18&30-VP1, VP1-III, or VP1-III-mRNA, respectively. *P < 0.05; **P < 0.01; ***P < 0.001; ****P < 0.0001.

Beyond humoral immunity, cellular responses are considered essential for viral clearance and long-term protection (17). Accordingly, antigen-specific T-cell responses induced by VP1-III and VP1-III-mRNA vaccines were examined. Molecular docking simulations performed with ClusPro 2.0 predicted stable binding interfaces between the epitopes of VP1-III and representative alleles of both MHC-I and MHC-II molecules, suggesting the potential to activate T-cell responses (Fig. S1). Splenocyte proliferation assays showed that all groups exhibited significantly higher proliferative responses following peptide stimulation, indicating activation of antigen-specific cellular immunity (Fig. 8A). No significant differences in proliferation were observed among the E11&18&30-VP1, VP1-III, and VP1-III-mRNA groups. Cytokine profiling of splenocyte supernatants revealed that IFN-γ production was significantly increased in all three immunized groups, indicating effective cellular immune activation (Fig. 8B). However, the magnitude of IFN-γ induction in the E11&18&30-VP1 group (113.21 pg/mL) was lower than that observed in the VP1-III (148.96 pg/mL) and VP1-III-mRNA (162.93 pg/mL) groups. A significant increase in IL-4 production was observed only in the VP1-III and VP1-III-mRNA groups, whereas no significant elevation in IL-4 levels was detected in the E11&18&30-VP1 group (Fig. 8C). Consequently, all three groups exhibited increased IFN-γ/IL-4 ratios, indicative of a Th1-biased immune response (Fig. 8D). These findings indicate that the mRNA platform may induce more functional and balanced immune responses.

## DISCUSSION

In this study, dominant B-cell epitopes within the VP1 proteins of E11, 18, and 30 were identified through bioinformatic prediction and ELISA screening, and incorporated into a chimeric trivalent antigen, VP1-III, via loop substitution. Both recombinant protein and mRNA formulations elicited robust cross-neutralizing antibody responses against all three serotypes in mice, with high-dose mRNA vaccination demonstrating superior potency. These results establish VP1-III as a promising trivalent vaccine candidate and underscore the advantages of mRNA-based platforms for multivalent enterovirus vaccine development.

Echoviruses have exhibited increasing prevalence in recent years, with multiple serotypes co-circulating globally; however, no licensed vaccines are currently available. Multivalent vaccine design strategies generally include (i) direct combination of serotype-specific antigens, (ii) epitope-based assembly guided by bioinformatic prediction, and (iii) loop substitution, where surface-exposed loops from different serotypes are rationally exchanged to create chimeric immunogens (18, 19). While the first approach is established but production-intensive, and the second enables computational design at the cost of conformational integrity, the third preserves native epitopes but requires extensive screening. Xia group recently exemplified this strategy through structure-guided loop grafting, producing chimeric HPV-VLP with broad cross-neutralizing activity (20). In this study, bioinformatic epitope prediction combined with a loop substitution strategy was used to develop a multivalent Echovirus vaccine. Predicted and screened immunodominant epitopes from E11, E18, and E30 were inserted into structurally defined VP1 loop regions, preserving conformational epitopes and generating a trivalent chimeric antigen, VP1-III, with broad protective potential. Immunization with VP1-III elicited antisera capable of neutralizing all three echoviruses; however, E11 required lower serum dilutions to achieve effective neutralization, whereas E18 and E30 were neutralized at higher dilution levels. This difference in neutralization efficiency may be partially explained by epitope distribution. Briefly, epitope mapping revealed that the VP1 protein of E11 contains fewer neutralizing epitopes than those of E18 and E30. In addition, the E11 strain used in this study displayed higher virulence relative to E18 and E30, which may partly explain why substantially lower serum dilutions were required to achieve effective neutralization against E11.

Compared with protein-based vaccines, mRNA platforms offer distinct advantages, including rapid design, scalable manufacturing, and potent immunogenicity (21, 22). For the corresponding mRNA vaccine, VP1-III-mRNA, low-dose immunization elicited minimal responses, whereas high-dose administration (10 µg/mouse) induced markedly higher antibody titers and stronger neutralizing activity against all three Echovirus serotypes relative to the recombinant protein formulation. This enhanced immunogenicity likely underscores the superior immunogenic potential of the mRNA platform for multivalent enterovirus vaccine development. In addition, all three groups elicited Th1-biased immune responses, as reflected by increased IFN-γ/IL-4 ratios. Notably, the E11&18&30-VP1 group exhibited relatively weaker cellular immune responses, characterized by a lower magnitude of IFN-γ induction and the absence of a significant increase in IL-4 production. This attenuated cellular response may stem from two complementary factors: first, antigenic competition and suboptimal epitope presentation inherent to physical mixtures of multiple proteins, which can reduce the efficiency of antigen processing and T-cell priming; second, dose dilution of serotype-specific antigens, as the total protein mass of the trivalent E11&18&30-VP1 mixture was matched to that of the chimeric VP1-III, resulting in each individual VP1 component being administered at a one-third lower dose, which may further dampen serotype-specific T- and B-cell activation. In contrast, the chimeric VP1-III antigen integrates multiple neutralizing epitopes within a single structural framework, potentially facilitating better antigen presentation and T-cell activation, thereby promoting stronger cellular immune responses at an equivalent total protein dose.

Furthermore, three conserved epitopes within the EF loop of E11-VP1, E18-VP1, and E30-VP1 were identified. Immunization of mice with the corresponding synthetic peptides revealed distinct immunogenic profiles. Notably, antisera raised against E11-Peptide 2 exhibited strong cross-reactivity toward all three peptides, whereas antisera against E18-Peptide 3 and E30-Peptide 4 exhibited limited cross-reactivity. Sequence alignment analysis indicated that the principal variations among these epitopes are located at residues 18–24, which may serve as critical determinants of serotype specificity (Fig. 3A). Building on these findings, we intend to design hepatitis B core-based virus-like particles (HBc-VLPs) displaying the three epitopes to further assess their potential as vaccine candidates. However, a major limitation remains the inherent resistance of mice to Echovirus infection, as no standardized *in vivo* model is currently available for efficacy evaluation, despite numerous studies employing IFN^−/−^ or hFcRn-IFN^−/−^ mice (23–25). Future studies will therefore aim to establish physiologically relevant animal models to rigorously assess the protective efficacy of these vaccine candidates.

This study demonstrates that a structure-guided loop substitution strategy can generate a trivalent Echovirus antigen (VP1-III) capable of inducing broad cross-neutralization and Th1-biased immune responses, and the VP1-III-mRNA (high dose) further enhanced immunogenicity. These findings establish a rational framework for developing multivalent vaccines against antigenically diverse enteroviruses and highlight the potential of mRNA platforms for rapid, broad-spectrum antiviral vaccine design.

## MATERIALS AND METHODS

### Cell lines and viruses

RD cells (CCL-136; ATCC) were cultured in high-glucose Dulbecco’s modified Eagle’s medium (DMEM; KeyGEN BioTECH, Jiangsu, China) containing 10% fetal bovine serum (FBS; ExCell Bio, Jiangsu, China) in a 37°C incubator containing 5% CO_2_. E11, E18, and E30 strains were obtained from stool samples of pediatric patients with Echovirus infection and amplified in RD cells. The accession numbers of E11, E18, and E30 were OR039176.1, OR039189.1, and OR039196.1, respectively.

### Cloning, expression, and purification of proteins

The VP1 genes of E11, E18, and E30 were derived from the strains above. All recombinant proteins (E11-VP1, E18-VP1, and E30-VP1) were expressed in *E. coli* BL21 (DE3) using pET-28a(+) expression plasmids. The coding sequences were codon-optimized for *E. coli* expression, and the resulting recombinant plasmids were synthesized by GenScript Biotech Co., Ltd. (Nanjing, China). A C-terminal His-tag was introduced to facilitate purification. Protein expression was induced with 1 mM IPTG at 18°C for 12 h in LB medium. Cells were harvested by centrifugation and resuspended in lysis buffer (50 mM NaH_2_PO_4_, 300 mM NaCl, 0.5 g/L lysozyme, and 1% Triton X-100). Cell disruption was achieved by ultrasonication on ice for 30 min, followed by solubilization in denaturing buffer (8 M urea, 50 mM NaH_2_PO_4_, 300 mM NaCl, and 10 mM imidazole). The denatured proteins were purified by Ni-NTA Beads (Smart-Lifesciences, China) and subsequently refolded by gradient dialysis. The chimeric construct (VP1-III) was designed based on E11-VP1, E18-VP1, and E30-VP1 sequences, and expressed using the same method as described above.

### Phylogenetic analysis

VP1 sequences from Echovirus serotypes were obtained from the NCBI GenBank database. Multiple sequence alignment was performed using MEGA11, and a phylogenetic tree was constructed based on the neighbor-joining method (26). The resulting tree was visualized and annotated using iTOL (https://itol.embl.de/index.shtml) (27). The Echoviruses VP1 protein sequences were downloaded from GenBank. E1: AF029859, E2: AY302545, E3: AY302553, E4: AY302557, E5: AF083069, E6: AY302558, E7: AY302559, E9: X84981, E12: X79047, E13: AY302539, E14: AY302540, E15: AY302541, E16: AY302542, E17: AY302543, E19: AY302544, E20: AY302546, E21: AY302547, E24: AY302548, E25: AY302549, E26: AY302550, E27: AY302551, E29: AY302552, E31: AY302554, E32: AY302555, and E33: AY302556.

### Prediction of B-cell epitopes

B-cell epitopes of E11, E18, and E30-VP1 were predicted using three independent online servers: ABCpred, BCPreds, and Bepipred Linear Epitope Prediction 2.0 (28–30). The parameters were set as follows: For ABCpred, peptide length = 16 aa, threshold = 0.5; For BCPreds: peptide length = 16 aa, threshold = 0.9; and For Bepipred 2.0: threshold = 0.5. Overlapping epitopes simultaneously predicted by all three tools were selected as candidate B-cell epitopes for each serotype.

### Prediction of three-dimensional structure and molecular docking

The three-dimensional structure of the VP1-III antigen was modeled using SWISS-MODEL, with the cryo-electron microscopy structure of VP1 (PDB ID: 6LA4; resolution: 2.34 Å) as the template (31). Molecular docking simulations between VP1-III and immune receptors MHC I and MHC II were performed using ClusPro 2.0 (32). The resulting docking poses were analyzed and visualized with BIOVIA Discovery Studio 2025 Client (33).

### Preparation and characterization of VP1-III-mRNA vaccine

The mRNA-lipid nanoparticle formulation was prepared following previously established protocols (34). VP1-III mRNA was synthesized from a linearized pUC19-VP1-III plasmid containing a 5′UTR, VP1-III coding sequence, 3′UTR, and a 100 nt poly(A) tail. Following linearization with *EcoRI*, capped mRNA was generated using the EasyCap T7 Co-transcription Kit with CAG Trimer (Vazyme, China). LNPs were prepared using SM-102, 1,2-distearoyl-sn-glycero-3-phosphocholine (DSPC), cholesterol, and 1,2-dimyristoyl-rac-glycero-3-methoxypolyethylene glycol-2000 (DMG-PEG2000) at a molar ratio of 50:10:38.5:1.5. Lipids dissolved in ethanol were combined with mRNA diluted in 50 mM sodium citrate (pH 5.0) using a microfluidic mixing system at a flow rate ratio of 3:1 and total flow rate of 1.3 mL/min. The mRNA-LNP was immediately diluted to less than 2% ethanol with PBS via ultrafiltration and concentrated. Particle size, polydispersity index (PDI), and zeta potential were measured with a ZetaPlus analyzer (Brookhaven, USA). Encapsulation efficiency was quantified with the Quant-iT RiboGreen RNA Assay Kit (Thermo Fisher, USA), and nanoparticle morphology was observed by transmission electron microscopy (H-7650, Hitachi, Japan).

### Production of polyclonal antibodies

Purified proteins (E11-VP1, E18-VP1, E30-VP1, VP1-III, or E11&18&30-VP1 group) were emulsified with equal volumes of Freund’s complete adjuvant for the primary immunization and Freund’s incomplete adjuvant for booster injections. Each group included six C57BL/6 mice (4–6 weeks old, male), which received 0.1 mg of antigen per dose by subcutaneous injection on days 0, 14, and 28. For the E11&18&30-VP1 group, equal amounts of E11-VP1, E18-VP1, and E30-VP1 were mixed, with each protein administered at 0.033 mg per dose (total antigen dose of 0.1 mg). For mRNA immunization, mice received intramuscular injections of VP1-III-mRNA formulated in LNPs at 1 µg (low dose) or 10 µg (high dose) per injection. Blood samples were collected retro-orbitally on days −1, 7, 21, and 35.

### Enzyme-linked immunosorbent assay (ELISA)

ELISA plates were coated overnight at 4°C with antigen (100 µg/mL, 100 µL/well). After washing with PBST, wells were blocked with 200 µL of blocking buffer for 2 h at 37°C. Diluted mouse sera (100 µL/well) were added and incubated for 2 h at 37°C, followed by HRP-conjugated goat anti-mouse IgG (1:5,000 dilution) for 1 h. After TMB substrate development for 30 min, the reaction was stopped, and absorbance was measured at 450 nm to determine antibody titers.

### Plaque reduction neutralization test (PRNT)

RD cells were seeded in 24-well plates and cultured to 80–90% confluence. Heat-inactivated mouse sera (56°C for 30 min) were serially diluted twofold and mixed with 50 PFU of Echovirus, followed by incubation at 37°C for 2 h. The mixtures were added to RD cells and incubated for 1 h. Cells were overlaid with 1% agarose and incubated for 72 h at 37°C. Following fixation and staining with 2% crystal violet, plaques were enumerated to determine neutralizing activity. The 50% plaque reduction neutralization titer (PRNT_50_) was defined as the reciprocal of the highest serum dilution that reduced the number of plaques by 50% compared with virus control wells.

### Viral neutralization by quantitative PCR

RD cells were cultured in 96-well plates and infected as described above using 200 TCID_50_/0.1 mL of Echovirus. After incubation for 48 h, total RNA was extracted, reverse transcribed, and analyzed using SYBR Green qPCR (Pro TaqHS Premix, High Rox Plus). GAPDH was used as an internal control. Viral RNA levels were quantified to determine the neutralization efficiency of different sera. E11-Prime-F: CAGTTGCTAGGGTAGCGGAC; E11-Prime-R: GTGTCCCCAGGTACCACTTG; E18-Prime-F: AACAGTCGCAAACACACAGC; E18-Prime-R: AGTTTGTAGGGTGTCGCTGG; E30-Prime-F: GAGACAGGGCACACATCACA; E30-Prime-R: CCGCCCTACCCATGAAGTTT. GAPDH-Prime-F: AGGTCGGTGTGAACGGATTTG; and GAPDH-Prime-R: TGTAGACCATGTAGTTGAGGTCA.

### Cellular immune response

On day 35 after immunization, mice were sacrificed, and spleens were aseptically collected. Splenocytes were isolated via mechanical dissociation and red blood cell lysis. Cells were seeded at 5 × 10^5^ cells/well in 96-well plates and stimulated with 10 µg/mL of peptide antigens for 72 h. Cell proliferation was assessed by the CCK-8 assay (20 µL/well, incubation for 2 h at 37°C, OD_490_ measurement). Supernatants were collected to measure IFN-γ and IL-4 levels by ELISA, and the IFN-γ/IL-4 ratio was calculated to evaluate Th1/Th2 polarization.

### Statistical analysis

Data are expressed as mean ± standard deviation (SD) and analyzed with GraphPad Prism 10. Group comparisons were performed using paired *t*-tests, one-way ANOVA, or two-way ANOVA with statistical significance defined as ns, *P* > 0.05; *P ≤ 0.05; **P ≤ 0.01; ***P ≤ 0.001; and ****P ≤ 0.0001.

## Data Availability

Data will be made available on request.

## SUPPLEMENTAL MATERIAL

Supplemental material jvi.00457-26-s0001.doc Table S1, predicted B cell epitopes; Fig. S1, molecular docking.

ASM does not own the copyrights to Supplemental Material that may be linked to, or accessed through, an article. The authors have granted ASM a non-exclusive, world-wide license to publish the Supplemental Material files. Please contact the corresponding author directly for reuse.
