# Supplementary material for: A chimeric trivalent Echovirus vaccine designed by loop substitution elicits cross-neutralizing immunity against serotypes 11, 18, and 30
Source: J Virol. 2026 May 5;100(6):e00457-26. doi: 10.1128/jvi.00457-26 (PMC13289168; doi:10.1128/jvi.00457-26)
Supplement: Supplemental material — Table S1, predicted B cell epitopes; Fig. S1, molecular docking. [file jvi.00457-26-s0001.doc]

**A chimeric trivalent Echovirus vaccine designed by loop substitution elicits cross-neutralizing immunity against serotypes 11, 18, and 30**

Shuai Jiang^a, 1^, Jiaqi Cui^a, 1^, Tao Sun^a^, Jiahuang Li^c^, Heming Li^a^, Tianlun Chen^a^, Jiahui Wu^a^, Zhenhua Zheng^d^, Jianbo Xia^b^, Chunchen Wu^b, **^, Jie Wu^a, *^

a School of Life Science and Technology, China Pharmaceutical University, Nanjing, 211198, China.

b Department of Laboratory Medicine, Maternal and Child Health Hospital of Hubei Province, Tongji Medical College, Huazhong University of Science and Technology, Wuhan, 430070, China.

c School of Biopharmacy, China Pharmaceutical University, Nanjing, 211198, China.

d CAS Key Laboratory of Special Pathogens and Biosafety, Center for Emerging Infectious Diseases, Wuhan Institute of Virology, Chinese Academy of Sciences, Wuhan, 430071, China.

* Corresponding author.

** Corresponding author.

*E-mail Addresses:* [wujie@cpu.edu.cn](mailto:wujie@cpu.edu.cn) (J. Wu), Chunchen Wu: chunchen_wu@126.com (C. Wu).

^1^ Shuai Jiang and Jiaqi Cui contributed equally to this work.

**Table S1.** Predicted B cell epitopes of Echovirus 11/18/30 VP1 proteins.

| **No.** | **Name** | **Sequence** | **Start** | **End** | **Score** |
| --- | --- | --- | --- | --- | --- |
| 1 | E11-VP1 | TFVITSKQDQGTQLGQ | 120 | 135 | 0.85 |
| 2 |  | LGQDMPPLTHQVMYIP | 133 | 148 | 0.85 |
| 3 |  | QVMYIPPGGPIPKSTT | 143 | 158 | 0.91 |
| 4 |  | GGPIPKSTTDYAWQTS | 150 | 165 | 0.89 |
| 5 |  | DYAWQTSTNPSIFWTE | 159 | 174 | 0.80 |
| 6 |  | AYSNFYDGWSHFSQNG | 191 | 206 | 0.69 |
| 7 |  | TLNNMGQLYMRHVNGP | 212 | 227 | 0.91 |
| 8 |  | NGPSPLPMTSTVRVYF | 225 | 240 | 0.85 |
| 9 |  | CQYINAPTVNFSSTNI | 256 | 271 | 0.91 |
| 10 | E18-VP1 | NFMGRAACVFMDQYKL | 60 | 75 | 0.8 |
| 11 |  | CVFMDQYKLNGEETST | 67 | 82 | 0.68 |
| 12 |  | KLNGEETSTDNFAVWT | 74 | 89 | 0.76 |
| 13 |  | CQDQGTQLEQDMPVLT | 121 | 136 | 0.79 |
| 14 |  | QLEQDMPVLTHQIMYV | 127 | 142 | 0.66 |
| 15 |  | THQIMYVPPGGPIPAK | 136 | 151 | 0.81 |
| 16 |  | GGPIPAKVDSYEWQTS | 145 | 160 | 0.92 |
| 17 |  | SYEWQTSTNPSVFWTE | 154 | 169 | 0.84 |
| 18 |  | TTLNAMGKLFVRHVNK | 206 | 221 | 0.81 |
| 19 |  | SSPHQITSTIRVYFKP | 222 | 237 | 0.83 |
| 20 |  | PRPPRLCPYINKGDVN | 245 | 260 | 0.79 |
| 21 |  | PYINKGDVNFVVTEVT | 252 | 267 | 0.61 |
| 22 | E30-VP1 | EKVNDELDRYTNWEIT | 82 | 97 | 0.71 |
| 23 |  | NWEITTRQVAQLRRKL | 93 | 108 | 0.84 |
| 24 |  | TSSQRTSTTYASDSPP | 125 | 140 | 0.84 |
| 25 |  | YASDSPPLTHQVMYVP | 134 | 149 | 0.82 |
| 26 |  | QVMYVPPGGPIPKSYE | 144 | 159 | 0.84 |
| 27 |  | GGPIPKSYEDFAWQTS | 151 | 166 | 0.92 |
| 28 |  | DFAWQTSTNPSVFWTE | 160 | 175 | 0.85 |
| 29 |  | PKHVKAWVPRAPRLCP | 243 | 258 | 0.88 |
| 30 |  | PYLYARNVNFDVQGVT | 258 | 273 | 0.65 |


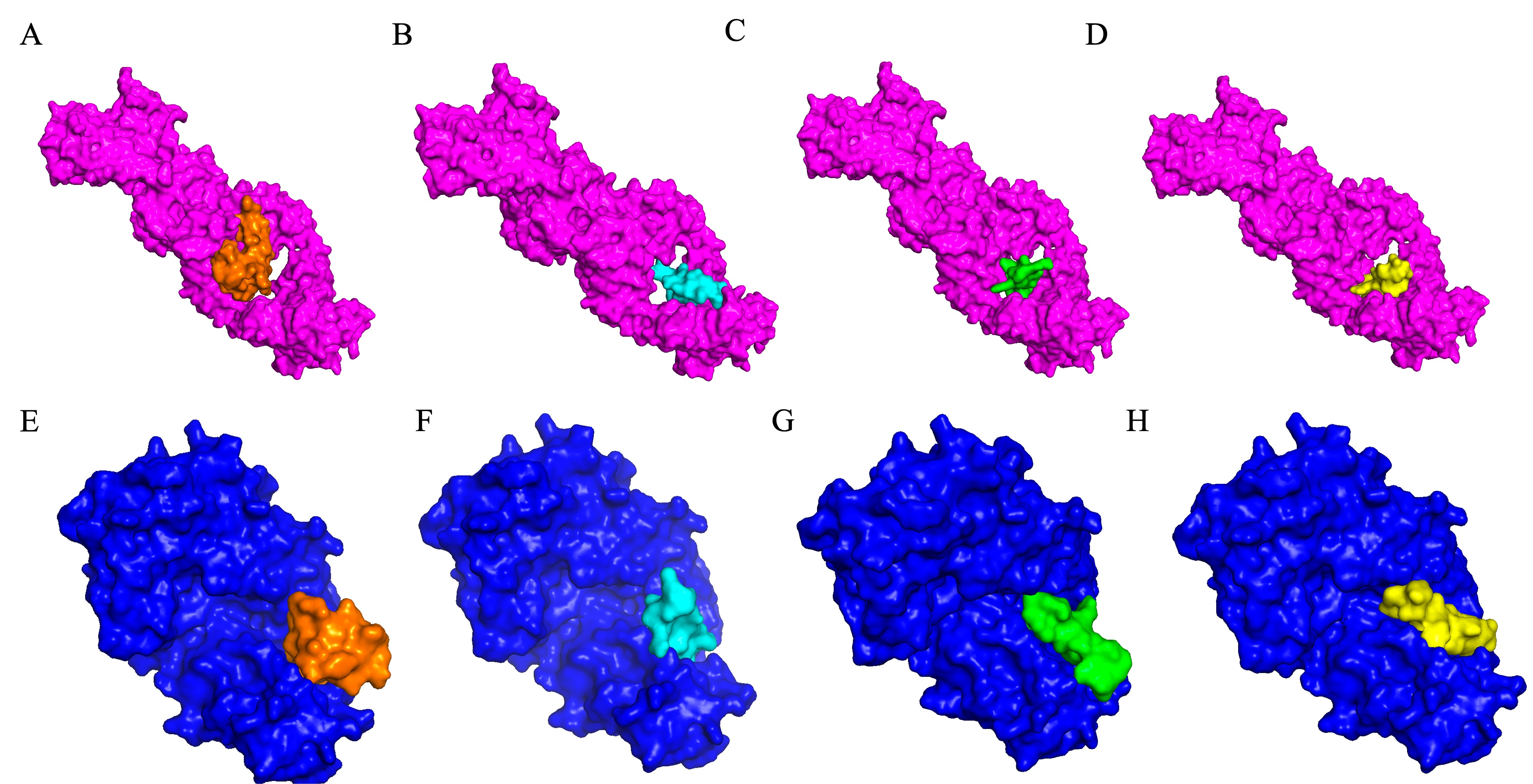


**Fig. S1.** Molecular docking of the E11-Peptide 2, E18-Peptide 2, E30-Peptide 1, and E30-Peptide 6 (Colored orange, cyan, green and yellow, respectively) with human immune receptors, including (A-D) MHC I (Colored pink) and (E-H) MHC II (Colored blue).
